# Rates of Spawning and Mortality Using Contrasting Methods for Culling Pacific Crown-of-Thorns Starfish, *Acanthaster* cf. *solaris*

**DOI:** 10.3390/biology14121720

**Published:** 2025-12-01

**Authors:** Morgan S. Pratchett, Ciemon F. Caballes, Leighton T. Levering, Deborah Burn, Josie F. Chandler, Alec S. Leitman, Peter C. Doll

**Affiliations:** 1 College of Science and Engineering, James Cook University, Townsville, QLD 4811, Australia; leighton.levering@my.jcu.edu.au (L.T.L.); josie.chandler@my.jcu.edu.au (J.F.C.); alecleitman@gmail.com (A.S.L.); peter.doll@jcu.edu.au (P.C.D.); 2 Marine Laboratory, University of Guam, Mangilao, GU 96923, USA; caballesc@triton.uog.edu; 3 Australian Institute of Marine Science, Townsville, QLD 4810, Australia; d.burn@aims.gov.au

**Keywords:** coral reefs, disturbance, culling, management, spawning, removal

## Abstract

Timely, concerted, and persistent culling (or manual removal) is required to effectively manage population irruptions of crown-of-thorns starfish (CoTS; *Acanthaster* spp.). However, there are concerns that handling and culling gravid starfish may induce spawning. This study explicitly tested the frequency and timing of spawning for Pacific CoTS (*Acanthaster* cf. *solaris*) injected with either bile salts (10 mL of 8 g·L^−1^ Bile Salts No. 3) or vinegar (20 mL of 4% acetic acid, with 10 mL injected into each of two non-adjacent arms), up to 48 h after treatment, while also considering three distinct experimental controls (handling controls, injection controls, and spawning controls). This study showed that male CoTS often spawn within 24 h after different culling treatments. However, the incidence of spawning by male starfish injected with vinegar (70%) was nearly twice that of male starfish injected with bile salts (36.4%). In contrast, there were no instances of spawning by female CoTS following handling or injections of bile salts and vinegar. Variation in the incidence of spawning between culling treatments is largely attributable to differences in the rate of mortality, whereby CoTS injected with bile salts (n = 23) consistently died within 24 h and therefore had limited opportunity to spawn. Meanwhile, CoTS injected with vinegar generally died >24 h post-treatment, and many had not died even after 48 h. This suggests that, where available, bile salts (rather than vinegar) should be used when culling *Acanthaster* cf. *solaris*, especially during reproductive periods. However, sustained culling effort is still the most direct and effective way to suppress the local densities and reproductive capacity of CoTS.

## Simple Summary

Crown-of-thorns starfish are a major cause of coral mortality and reef degradation, especially within coral reef regions that are subject to recurrent population irruptions (or outbreaks) of these starfish. Accordingly, there are extensive and ongoing efforts to suppress densities of crown-of-thorns starfish, either by injecting individual starfish with toxic chemicals or manually removing them from reef environments. There are concerns, however, that these management actions may inadvertently cause adult starfish to reproduce, ultimately adding to the problem. This study showed that handling and injecting crown-of-thorns starfish during peak reproductive periods may cause them to release their gametes, though this only applies for male starfish, and this effect also varies depending on the chemical being used. While it may be counter-beneficial to completely halt culling and removal programs during the extended breeding seasons of crown-of-thorns starfish, this study does provide guidance on how best to reduce the risks, of unintended consequences, which may otherwise perpetuate high densities of crown-of-thorns starfish.

## 1. Introduction

Crown-of-thorns starfish (CoTS; *Acanthaster* spp.) are native to coral reefs throughout the Indo-Pacific [1,2], but are regarded as a pest, given their tendency to undergo population irruptions, also referred to as plagues [3] or outbreaks [4], which can cause extensive damage to coral reef ecosystems [5,6,7,8]. Adult CoTS feed primarily on hard (order Scleractinia) corals [2,8,9,10] that are fundamental in providing habitat structure and supporting the exceptional diversity of marine species that associate with coral reefs [11]. Population irruptions of CoTS can cause extensive and widespread coral loss [6,7,8], and have been a major contributor to sustained reef degradation [12,13], especially in locations with persistent or recurrent population irruptions (e.g., Southern Japan [14], Australia [15], Moorea [16]). While there is no evidence that population irruptions of CoTS are becoming more or less frequent [10], the cumulative frequency and severity of major acute disturbances is increasing throughout the Indo-Pacific, primarily due to the escalating incidence of severe marine heat waves and corresponding mass coral bleaching [16,17,18]. These disturbances are reducing opportunities for coral reef recovery during the increasingly restricted periods between successive disturbances [18], while chronic pressures constrain rates of coral recovery [19,20]. A critical management priority is therefore to reduce coral loss caused by major disturbances, including population irruptions of CoTS [21].

The first hypotheses put forward to explain population irruptions of CoTS in the 1960s and 1970s tended to be predicated on the assumption that these were new and unprecedented phenomena linked to increasingly pervasive anthropogenic threats [9,22], such as coastal development [7], pesticides and pollutants [23], or over-fishing [24]. There are, however, conspicuous anecdotal reports of high densities of *Acanthaster* spp. from several locations across the Indo-Pacific from well before population irruptions were reported in the 1960s [3]. CoTS are also predisposed to major fluctuations in abundance given their inherent life-history characteristics [25,26,27,28]. While debate continues on whether population irruptions of CoTS are caused or exacerbated by anthropogenic activities [29,30], it is clear that coral reefs cannot withstand current and increasing levels of disturbances and pressures [13,17]. Moreover, anthropogenic pressures (e.g., fishing and harvesting, sedimentation, eutrophication, and pollutants) may have undermined the capacity of reef ecosystems to withstand perennial disturbances [10]. Accordingly, there are significant and widespread efforts to suppress population irruptions of CoTS and thereby minimize coral loss and reef degradation [14,21,30,31,32]. Population irruptions of CoTS are also more amenable to direct management intervention [32,33], compared to other major disturbances (e.g., climate-induced coral bleaching and severe tropical storms).

Effective and enduring management of CoTS depends on understanding the factor(s) that initiate or exacerbate population irruptions [29]. However, critical research is still required to firmly establish the underlying causes of population irruptions [5,27,34,35]. It is also likely that population irruptions are caused by the confluence of multiple factors [29,36], some of which cannot be managed or controlled [32]. Meanwhile, manual removal or culling (using in situ injections of toxic chemicals) of individual CoTS remains the most direct and effective mechanism to reduce local densities and contain population irruptions [31,32]. The Australian Government has invested >AUD100 million in culling CoTS on the Great Barrier Reef (GBR), which is considered central to ecosystem resilience [21]. The CoTS control program on the GBR was first initiated in 2002, with intensive culling being undertaken in the immediate proximity of major tourism infrastructure and using sodium bisulphate solution, which required multiple injections in the individual arms and oral disk of each starfish [21]. Greatly increased efficiency and effectiveness of CoTS control were achieved in the 2010s with the introduction of single-shot injection methods, whereby CoTS were injected with lethal doses of bile salts [37], which facilitated major expansion in the level and extent of control activities on the GBR [21]. There has since been demonstrated capacity to suppress CoTS densities following the establishment and detection of population irruptions at individual reefs [32] and across regions [21].

Timely, concerted, and persistent culling effort is required to effectively reduce elevated densities of CoTS [21,31,32]. For example, Westcott et al. [32] estimated that >15 separate culling episodes are required to effectively redress localized infestations of crown-of-thorns and prevent coral loss, though successful management was mainly achieved inside no-take reserves. Despite the need for persistent culling over multiple years, some researchers have cautioned against disturbing CoTS during reproductive periods [31]. Removing or culling CoTS before they spawn will help minimize the risk of prolonging or spreading population irruptions [31]. However, there are concerns that culling during reproductive periods may unintentionally induce spawning [38,39]. For example, Dumas et al. [39] suggested that the removal of CoTS may trigger synchronized spawning under certain temporal and spatial conditions, as evidenced by gonadosomatic index values of starfish from culled areas being consistently around half those from control sites. Subsequent research suggested that injecting CoTS with vinegar posed limited risk of initiating mass-spawning [40]. However, these concerns remain unverified through rigorous empirical testing. On the GBR, Pacific CoTS (*Acanthaster* cf. *solaris*) spawn mainly in late December [41], though the timing and extent of spawning synchrony may vary greatly, depending on seasonal changes in water temperature [42]. Caballes and Pratchett [43] suggested that environmental cues (especially temperature change) were important for coordinating reproductive activities, but spawning is mainly induced by the initial release of sperm. Consequently, if male CoTS release sperm due to collection or culling during peak reproductive periods, this may initiate localized spawning of gravid conspecifics that have not been collected or culled.

The purpose of this study was primarily to test the likelihood that current culling methods used on Australia’s GBR (namely, injecting with either vinegar or bile salts [21]) will unintentionally induce spawning. Experimental administration of toxic chemicals was conducted in semi-closed systems to prevent the release of gametes into the environment, necessitating extensive handling of individual starfish (during collection and re-location to experimental facilities) ahead of experimental treatments. Accordingly, there was further opportunity to assess whether the manual removal of CoTS during peak reproductive periods would potentially induce spawning. Moreover, starfish were held for up to 48 h after the experimental treatments, which allowed for direct comparisons of the effectiveness of different culling methods by comparing the rates of mortality.

## 2. Materials and Methods

This study was conducted at Lizard Island in the northern GBR, Australia. A total of 76 *A.* cf. *solaris* (20–48 cm diameter) were collected from reefs (mainly Martin and Eyrie Reefs) located within 10 km of Lizard Island. There was no discrimination based on size or sex when collecting starfish, rather, we systematically searched along the reef on SCUBA and collected all individuals that were detected. Individual CoTS were collected using large tongs and placed into a large collection bag for transport to the nearby research vessel, before being placed into large (70 L) tubs filtered with fresh seawater for transport to the research station. Care was taken not to damage the starfish during collection and transport. The time between collection and the commencement of planned experiments ranged between 4 and 10 h, reflecting the time required to collect and process starfish. During this period, starfish were monitored for any signs of spawning, and any starfish that spawned prior to the commencement of the experiment were not used in subsequent experiments. Only one individual, a large male starfish (53 cm diameter) was observed to spawn spontaneously upon initial disturbance, even before being placed in the collection bag.

Upon arrival at the Lizard Island Research Station, CoTS were transferred to the aquarium facility, where each individual was examined to determine sex and measure size. Sex was determined by extracting a small sample of gonad material using a biopsy needle, which was then placed in fresh water to induce gamete release, whereby males and females were identified based on presence of sperm versus eggs, respectively. The size of CoTS was measured based on their maximum diameter (cm). Each starfish was placed in an individual 42 L tub and allocated to an appropriate treatment. All starfish contained readily distinguishable gametes, and though there was no accounting for individual reproductive condition, it was also apparent that many of the starfish were highly gravid, especially the larger females, with highly distended tissues at the upper extent of arms. Individual starfish were held in experimental tanks for a minimum of 1 h before experimental treatments were imposed.

The experimental tubs were contained within an even larger (1.8 m diameter) circular trough (5 tubs per trough), to contain any gametes that were released during the experiment. We maintained a continuous, low flow of fresh seawater (20 L·h^−1^) to each of the tubs throughout the experiment, but also completely flushed tubs using unfiltered seawater every 12 h to counter potential de-oxygenation, especially after major spawning. Unfiltered seawater was pumped directly from the sea (temperature = 27.8 °C, salinity = 33 ppt) into a large header tank before being gravity-fed to each tub. Adult CoTS were collected on three separate occasions (28 November, 30 November, and 2 December 2023), with experiments run over three separate trials (starting 15:20 h on 28 November, 14:00 h on 30 November and 10:30 h on 3 December 2023). All tubs were emptied and thoroughly washed with fresh water between trials. All replicates were effectively independent, and each individual starfish was used in only a single trial and a single treatment. All starfish that were still alive at the termination of each trial were injected with bile salts and then buried.

The primary objective of these experiments was to directly compare rates of spawning and mortality for *A.* cf. *solaris* injected with either bile salts (10 mL of 8 g·L^−1^ Bile Salts No. 3 [37], New Zealand Pharmaceuticals (NZP), Palmerston North, New Zealand), administered as 5 mL doses into the base of each of two non-adjacent arms) or vinegar (20 mL of 4% acetic acid [44], administered as 10 mL doses into the base of each of two non-adjacent arms). Accordingly, the majority of starfish were assigned to these treatments (Table 1). There were also three different control treatments, including (i) handling controls—whereby starfish were not manipulated any further after being placed in the experimental tubs, (ii) injection controls—where starfish were injected with 10 mL of distilled water (administered as 5 mL doses into the base of each of two non-adjacent arms), and (iii) positive spawning controls—where starfish were injected with 4 mL of 1-methyladenine (1-ma) solution [45] administered as 1 mL doses into each of 4 non-adjacent arms. The latter was used to confirm that CoTS used in these experiments were capable of spawning.

Experiments were conducted as soon as practical after all CoTS had been collected and processed, allowing for a minimum of 1 h (and up to 15 h) to become acclimatized to the experimental tubs before administering relevant treatments. Each of the various treatments were administered using separate injecting apparatus (Simcro plastic syringes fitted with Luer-lock needle holders and a 16G needle [37]) to minimize risk of cross-contamination. Starfish were observed continuously for the first 60 min to assess the time until gametes started being released. Thereafter, observations were conducted every 1–4 h, recording the earliest time at which spawning was observed and/ or gametes were detected in the water. The time to mortality was also recorded, with mortality presumed to have occurred when starfish were completely motionless, none of the podia (tube feet) were adhered to the plastic tub, and there was no response to tactile stimulation of podia.

### Data Analyses

Responses of CoTS (both in terms of spawning and mortality) to different treatments were compared based on both the frequency and rate. Frequency analyses were conducted using Generalised Linear Models (GLM) testing for independent effects of treatment and sex, as well as an interaction modeled against a binomial distribution with a log-link function (using R version 4.4.2 [46]). Deviance statistics were used to assess the significance of different effects. Relevant differences in the frequency of events were displayed using simple bar plots. To assess variation in the rate of spawning versus mortality, the time elapsed until specific events were recorded (in hours) and assessed using Kaplan–Meier curves and log-rank tests to compare among treatments. Kaplan–Meier estimates were generated per treatment, though all control treatments (handling, injection and spawning) were combined for the analysis of mortality (but not spawning), and plotted using the “survminer” [47] and “survival” packages [48]. Log-rank tests were conducted using the “survival” package. For spawning, curves show increases in the spawning probability of starfish up until a maximum of 48 h, based on the first evidence of spawning. There was no accounting for the extent or duration of spawning exhibited by each individual CoTS. For mortality, the curves show declines in the survival probability of starfish up until a maximum of 48 h.

## 3. Results

### 3.1. Spawning

**Figure 1:**
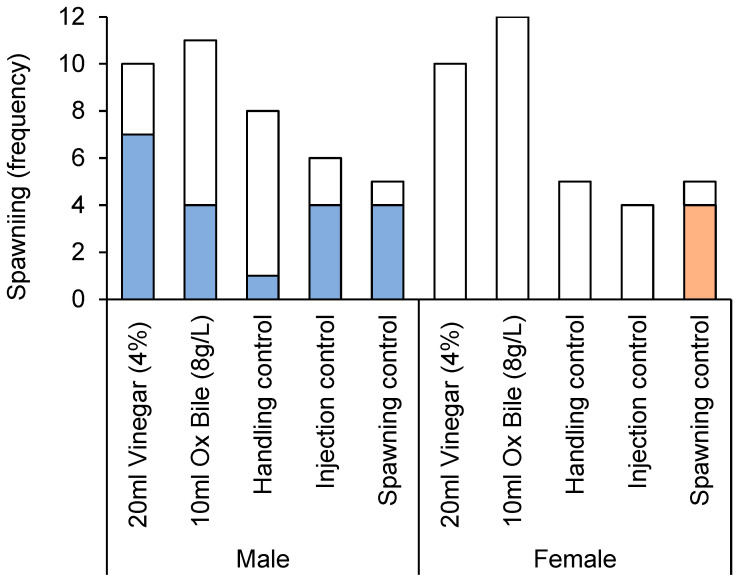
Spawning incidence of male versus female Pacific CoTS (*Acanthaster* cf. *solaris*) administered with bile salts (10 mL of 8 g·L^−1^ Bile Salts No. 3) or vinegar (20 mL of 4% acetic acid), versus three different controls, handling controls (no injections), injection controls (10 mL of distilled water), and spawning controls (4 mL of 1-ma). Colored bars indicate the number of starfish that spawned in each treatment, while white bars indicate the number that did not spawn.

High incidence of spawning was recorded among male CoTS during experimental studies to test different culling treatments. In all, 50% (20 out 40) of males used in this experiment were recorded to spawn, which was initially visible as discrete, fine lines of sperm being released, before the water within the relevant tub appeared cloudy. The highest incidence of spawning (80%) was recorded among males that were injected with 1-methyladenine (positive spawning controls), though the incidence of spawning for males injected with vinegar was also very high (70%). Overall, it appeared that injecting male starfish (whether using vinegar, bile salts, or distilled water) substantially increased the likelihood of spawning over and above handling (Figure 1). Even accounting for the single starfish that started spawning during initial collection, the overall incidence of spawning for male starfish that had not been injected (accounting for the often-extensive period up until the initiation of experiments) was <5% (2 out of 41).

The incidence of spawning was significantly different among treatments, but also varied with sex (Table 2). The incidence of spawning for male CoTS injected with vinegar (70%, 7 out 10 individuals) was nearly twice that of male starfish injected with bile salts (36.4%, 4 out of 11 individuals). Notably, none of the females spawned, except when specifically induced using 1-ma (Figure 1).

**Figure 2:**
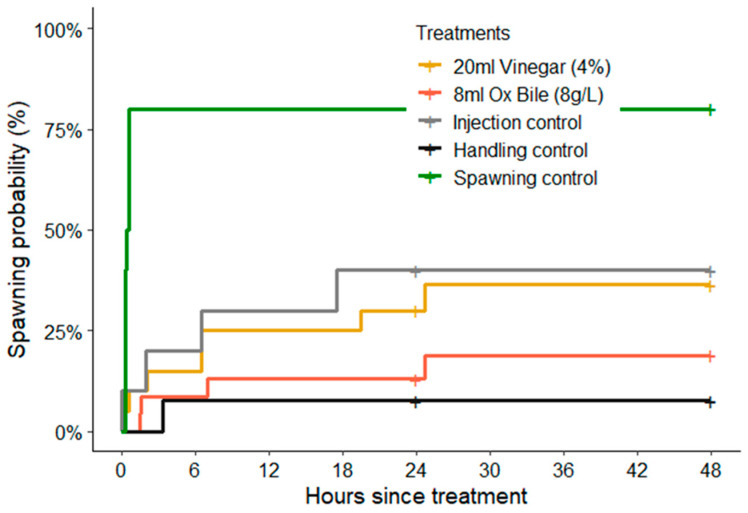
Cumulative spawning probabilities based on Kaplan–Meier estimates, showing overall percentage of CoTS (*Acanthaster* cf. *solaris*) that spawned in each experimental treatment. Data was pooled among sexes, though it was only male starfish that were recorded to spawn in response to handling and injection treatments (see Figure 1). The only recorded incidence of female spawning was following injections of 1-ma (positive spawning controls).

The rates (timing) of spawning were also significantly different among treatments (log-rank test: χ^2^ = 21.1, df = 4, *p* < 0.01), though this was mainly attributable to the consistently rapid initiation of spawning for CoTS that spawned after being injected with 1-methyladenine (within 15–35 min, Figure 2). For starfish injected with vinegar and bile salts, spawning was recorded at various intervals up to 24 h following treatments. There was also no obvious diurnal pattern in the timing of spawning, whereby starfish injected with vinegar spawned throughout the day (from 06:00 h to 16:05 h) and only two of the seven starfish spawned at night (22:00 h).

### 3.2. Mortality

**Figure 3:**
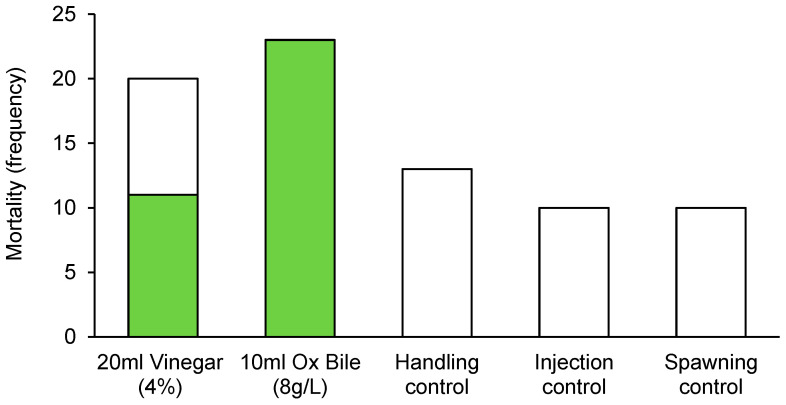
Incidence of mortality recorded for Pacific CoTS (*Acanthaster* cf. solaris) administered with bile salts (10 mL of 8 g·L^−1^ Bile Salts No. 3) or vinegar (20 mL of 4% acetic acid), versus three different controls, handling controls (no injections), injection controls (10 mL of distilled water), and spawning controls (4 mL of 1-ma). Colored bars indicate the number of starfish that had died by the end of the experiment, while white bars indicate the number that were still alive.

The incidence of mortality for CoTS was highly contingent upon culling treatment, with no mortality recorded for starfish in any of the control treatments (Figure 3). In contrast, there was 100% mortality among starfish (23 out of 23 individuals) injected with bile salts (10 mL 8 g·L^−1^ Bile Salts No. 3). For starfish injected with vinegar (20 mL of 4% acetic acid) 11 out of 20 (55%) starfish died within 48 h. While incidence of mortality was significantly different among treatments, there was no effect of sex (Table 2).

**Figure 4:**
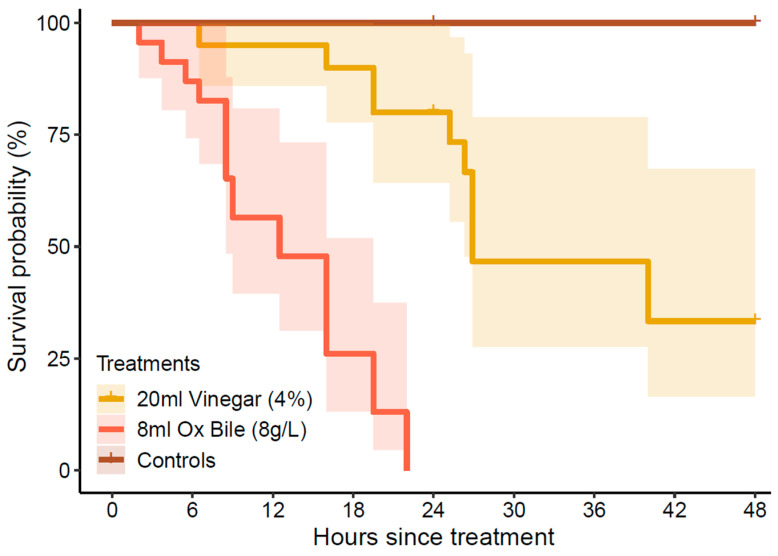
Cumulative mortality probabilities based on Kaplan–Meier estimates, showing the overall percentage of CoTS (*Acanthaster* cf. *solaris*) that died within 48 h after being administered with bile salts (10 mL of 8 g·L^−1^ Bile Salts No. 3) or vinegar (20 mL of 4% acetic acid). No mortality was recorded in any of the control treatments (see Figure 3). Shaded ribbons represent 95% confidence intervals.

The rate (timing) of mortality was significantly different among treatments (log-rank test: χ^2^ = 92.5, df = 4, *p* < 0.01), though this is partly attributable to the lack of mortality recorded across all control treatments. Even so, mortality of starfish injected with bile salts consistently occurred within 24 h of treatment, whereas starfish injected with vinegar generally survived for >24 h (Figure 4). Only 3 (out of 20) CoTS injected with vinegar that died inside of 24 h, including 1 male starfish that died almost immediately after spawning (6.5 h after treatment) and 2 highly gravid females that died 16–19 h after treatment). In the bile salts treatment, the first starfish that died (2.0 h after treatment) was a male that spawned (1.6 h after spawning), followed by a highly gravid female that died 3.7 h after treatment.

## 4. Discussion

This study showed that gravid male CoTS may begin spawning following handling and especially culling treatments. The number of *A.* cf. *solaris* that spawned in response to handling (independent of, or before, administration of culling treatments) was minimal (2 out of 41), even though the extent of handling to collect, transport, and establish individual starfish in experimental tubs was extensive and prolonged, especially compared to the time and handling required simply to remove the starfish from the marine environment [31,39]. The incidence of spawning increased markedly following the administration of injections, where even injection controls (10 mL of distilled water) caused extensive spawning among male *A.* cf. *solaris*. However, effects of the culling treatments and handling are additive (i.e., it was not possible to administer culling treatments under experimental conditions without first collecting CoTS). Notably, only male CoTS spawned, even though females were highly gravid, and responded rapidly to injections of 1-methyladenine, which is a gonad stimulant [45].

The time to spawning recorded for CoTS injected with vinegar, bile salts, or distilled water, was substantially longer and much more variable than starfish injected with 1-methyladenine. This suggests that these chemicals (unlike 1-methyladenine) do not directly stimulate gamete release. Rather, these starfish may be spawning in response to the physiological stress imposed by the chemicals, including the distilled water that would immediately reduce the internal salinity at sites of injection. Stress-induced spawning is well established among echinoderms and is often exploited to obtain gametes of cultured species [49], by physically or chemically disrupting the gonad wall and smooth muscle, thereby facilitating the release of gametes. It has also been suggested that some organisms will invest all remaining energy in reproduction when mortality is inevitable, in an attempt to further their genetic legacy [50]. In our study, there was limited evidence that spawning incidence was necessarily linked to impending mortality; out of the 34 starfish that died following the administration of vinegar or bile salts, only 7 spawned beforehand. Moreover, nine starfish that spawned (not including those injected with 1-methyladenine) did not subsequently die. In the two instances where spawning and mortality were highly coincident, there were very high levels of sperm release, and the subsequent deterioration of water quality may have compounded upon the effects of culling treatments to expedite mortality. This suggests that spawning occurs in direct response to extrinsic processes, and is largely independent of mortality, though increased rates of mortality will inevitably constrain opportunities for spawning.

The high incidence of spawning for CoTS administered with chemical injections partly validates concerns raised by some conservation organizations [51] that have advised against using chemical injections, even though they acknowledge the increased efficiency of in situ culling compared to manual removals. The critical question, however, is whether the risk posed by culling during reproductive periods justifies halting or constraining the necessary removal of CoTS to prevent their direct effects on coral assemblages and reef habitats. Notably, the timing of reproduction by CoTS is not known for many regions, and may be highly protracted [42], making it challenging to avoid culling during peak spawning periods. Meanwhile, the effectiveness of culling programs is invariably linked to the timeliness, intensity, and duration of culling effort [21,30,31]. While suspending culling during reproductive periods will not necessarily prevent local spawning, intensive and well-timed culling that prioritizes the removal of large, highly fecund individuals before they spawn remains the most effective strategy for constraining local reproductive output [52]. Reducing the local density, and therefore proximity of starfish, will also reduce fertilization success [53,54].

The risk associated with unintentionally inducing spawning by CoTS during handling or culling, is that gamete release into the marine environment will initiate a cascade of events that leads to increased reproductive success, thereby compounding or spreading population irruptions. Critically, only males were induced to spawn using culling injections in our experiments; however, the release of sperm may induce other male CoTS to spawn, leading to even higher concentrations of sperm that subsequently induce females to spawn [43]. Highly synchronized spawning among males and females in areas with high densities of CoTS will then result in high rates of fertilization success [53,54] that ultimately lead to increased rates of larval settlement on natal or nearby reefs [55,56]. While handling or culling activities will not necessarily cause or exacerbate localized spawning, it may be prudent to consider specific culling methods that reduce the likelihood of spawning without constraining the effectiveness of management programs intended to suppress local populations of CoTS.

### Contrasting Effectiveness of Culling Methods

Marked differences in the effectiveness of bile salts versus vinegar for culling CoTS were revealed during the current experiments, though experimental trials were run for a maximum of 48 h and starfish injected with vinegar may have died if experiments were run for another 24 h [44]. In previous experimental trials, the average time to mortality for *A.* cf. *solaris* injected with 10 mL of 8 g·L^−1^ Bile Salts No. 3 (the same volume, concentration and type of bile salts used in the present study) was 24 h [37]. In comparison, Boström-Einarsson et al. [44] reported an average time to mortality of 54 h for *A.* cf. *solaris* injected with 20 mL of 4% acetic acid, as used in the current experiments. However, the time to mortality following vinegar injections could be reduced by increasing the number and/ or volume of injections into the same starfish [37,44,57]. Increasing concentrations of vinegar appeared to reduce effectiveness in some trials [44], but produced relatively rapid and comprehensive mortality in others [57].

Previous tests of the effectiveness of different culling treatments have focused mainly on achieving comprehensive (100%) mortality, with limited consideration of time to mortality. Comprehensive mortality of *A.* cf. *solaris* has been achieved using 20 mL of 4% acetic acid both in laboratory experiments [58] and field trials [44]. However, there have been limited attempts to verify the effectiveness of different culling treatments under normal operating conditions, especially for large-scale programs. Specifically, the incidence and rate of mortality should be measured in the field by tracking the individual fate of CoTS known to have been subject to specific culling treatments. It is known, for example, that even slight changes in culling methods can greatly constrain effectiveness [44] and CoTS have remarkable capacity to survive and regenerate even if only a small portion of the original starfish persists [59]. The surest way to ensure that individual starfish do not cause any further damage to coral reef ecosystems is manual removal, though in situ culling requires much less time and effort and may allow for many more starfish to be effectively removed, depending on local densities [60] and detectability [61]. Current culling operations on the GBR use either bile salts or vinegar, depending on the availability of bile salts and the density of CoTS [21]. Unlike bile salts which degrade within days of being mixed in solution and also start to precipitate and clog the needle and injector mechanisms, vinegar can be stored for extended periods, which is especially useful when treating only limited numbers of CoTS each dive.

Our study shows that variation in the time to mortality among different culling treatments directly affects the opportunity for CoTS to spawn. The timing of spawning for male starfish injected with bile salts was not different from those injected with vinegar. However, given the time to mortality was much shorter using bile salts, there was much less opportunity for the starfish to spawn. This suggests that there may be benefits in optimizing culling treatments to minimize the time until mortality, so long as it does not compromise effectiveness. On the GBR, for example, culling operators do not suspend or reduce activities during the reproductive period, though bile salts were used exclusively throughout the 2024–2025 spawning season (following prompt communication of the results from these experiments), whereas vinegar is widely used at other times throughout the year.

## 5. Conclusions

Population irruptions of CoTS may or may not be caused by anthropogenic activities [29], but are undoubtedly contributing to sustained coral loss and reef degradation [7,8,9,10]. Extensive culling of CoTS is therefore justified to minimize ongoing coral loss [5,6] and effectively offset other distal drivers of coral reef degradation (e.g., environmental change) that are not amenable to direct and localized management [62,63]. Demonstrated effectiveness of large-scale culling programs [21] is also likely to further increase culling investment and effort. It is therefore important to ensure that this extensive management activity is not having perverse and unintended outcomes, which may ultimately worsen the outlook for coral reef ecosystems. This study demonstrates that spawning can be induced following culling treatments (at least among males), particularly when using vinegar injections, though this will necessarily initiate mass-spawning [57]. Overall, bile salts injections resulted in more rapid mortality compared to vinegar, thereby significantly reducing the window of opportunity for CoTS to spawn after treatment. As such, where bile salts are available and logistically feasible, it might be the preferred option for culling CoTS, particularly during peak spawning seasons, to minimize unintended gamete release and thereby limit the risks of unintentionally exacerbating population irruptions. However, it is important to emphasize that the risk of inducing spawning should not justify deferring or delaying culling activities. The direct and immediate threat posed by high densities of CoTS to coral reef ecosystems outweighs the relatively minor additional risk of spawning induction [57]. Maintaining sustained and intensive culling efforts remains critical to reducing both adult densities and overall reproductive output, as well as in mitigating ecological effects, even if some limited spawning occurs as an incidental outcome.

Culling CoTS represents one of the most direct and effective management strategies to minimize coral mortality but is unlikely to deliver long-term ecological benefits in isolation [63]. There may be further opportunities to increase the feasibility and effectiveness *A.* cf. *solaris* control, using novel methods and new technologies, and improving strategic allocation for management efforts [62,63]. However, permanent or long-term solutions will require a deeper, more holistic consideration of the multitude of factors that contribute to population irruptions [6]. Moreover, improvements in localized and direct management of the direct causes of coral mortality need to be accompanied by immediate and rapid reductions in global carbon emissions.

## Figures and Tables

**Table 1:** Overall sample size and average (and SE) size (diameter in cm) of male and female CoTS used in different experimental treatments.

	Females			Males		
Treatments	n	Size	SE	n	Size	SE
Bile salts (10 mL 8 g·L^−1^ bile salts)	12	36.00	1.49	11	33.18	1.90
Vinegar (20 mL of 4% acetic acid)	10	34.60	1.38	10	36.00	1.80
Injection control (10 mL distilled water)	4	34.38	2.08	6	36.67	2.99
Handling control	5	31.40	1.36	8	31.38	2.62
Spawning control (4 mL 1-ma)	5	32.20	1.16	5	31.40	1.72
Total	36	34.26	0.73	40	33.83	1.01

**Table 2:** Analysis of deviance for binomial GLM to test whether the differences in the frequency of (a) spawning and (b) mortality differed with treatment and sex.

Response	Terms	Deviance	df	*p*
(a) Spawning	Treatment	17.12	4	<0.01
	Sex	21.37	1	<0.01
	Treatment × Sex	5.99	4	0.20
(b) Mortality	Treatment	76.99	4	<0.01
	Sex	0.20	1	0.65
	Treatment × Sex	0.00	4	1.00

## Data Availability

Experimental data showing frequency and timing of spawning versus mortality are openly accessible via the JCU Research Data Hub (https://researchdata.jcu.edu.au/default/rdmp/home, accessed on 5 November 2025), or from the corresponding author upon request.

## Abbreviations

The following abbreviations are used in this manuscript:

CoTS	Crown-of-thorns starfish
GBR	Great Barrier Reef
1-ma	1-methyladenine
